# A Magnetically Actuated Superhydrophobic Ratchet Surface for Droplet Manipulation

**DOI:** 10.3390/mi12030325

**Published:** 2021-03-19

**Authors:** ChangHee Son, BingQiang Ji, JunKyu Park, Jie Feng, Seok Kim

**Affiliations:** 1 Department of Mechanical Science and Engineering, University of Illinois at Urbana-Champaign, Urbana, IL 61801, USA; cs21@illinois.edu (C.S.); jbq@illinois.edu (B.J.); jpark323@illinois.edu (J.P.); jiefeng@illinois.edu (J.F.); 2 Department of Mechanical Engineering, Pohang University of Science and Technology, Pohang 37673, Korea

**Keywords:** ratchet surface, superhydrophobic, Laplace pressure, droplet manipulation, NdFeB

## Abstract

A water droplet dispensed on a superhydrophobic ratchet surface is formed into an asymmetric shape, which creates a Laplace pressure gradient due to the contact angle difference between two sides. This work presents a magnetically actuated superhydrophobic ratchet surface composed of nanostructured black silicon strips on elastomer ridges. Uniformly magnetized NdFeB layers sputtered under the black silicon strips enable an external magnetic field to tilt the black silicon strips and form a superhydrophobic ratchet surface. Due to the dynamically controllable Laplace pressure gradient, a water droplet on the reported ratchet surface experiences different forces on two sides, which are explored in this work. Here, the detailed fabrication procedure and the related magnetomechanical model are provided. In addition, the resultant asymmetric spreading of a water droplet is studied. Finally, droplet impact characteristics are investigated in three different behaviors of deposition, rebound, and penetration depending on the impact speed. The findings in this work are exploitable for further droplet manipulation studies based on a dynamically controllable superhydrophobic ratchet surface.

## 1. Introduction

Liquid droplet manipulation has been popularly studied to satisfy the needs of various applications such as surface cleaning [1,2,3], chemical reactions [4,5], bioassays [6], and biosensing [7]. The methods to manipulate liquid droplets are commonly based on controlling the electric field, temperature, magnetic field, light, and surface wetting, together with passive activation via superhydrophobic surfaces [8,9,10]. In parallel to such methods, other droplet manipulation methods employing the asymmetric characteristics of ratchet surfaces have also received much attention.

For example, when a liquid droplet is dispensed onto a heated solid surface whose temperature is higher than the Leidenfrost temperature, it creates a thin film of vapor between the droplet and the surface. This vapor levitates and manipulates the droplet frictionlessly. Linke et al. found that this Leidenfrost effect can make a droplet self-propelled if it is placed on a ratchet surface [11]. The droplet can accelerate up to 1–2 m/s^2^ due to a viscous force that is generated by vapor flow through a gap between the droplet and the asymmetric ratchet surface. Ok et al. implemented a hydrophobic coating on ratchet surfaces, which increased the velocity of the droplet and decreased the threshold temperature of the droplet motion [12]. The direction of the droplet is also switchable by adjusting the topology of the rachet surfaces [13,14,15].

While there are plenty of studies of droplet manipulation based on the ratchet surface combined with the Leidenfrost phenomenon, other researchers also manipulated droplets on a rachet surface by applying vibration to the surface [1,16,17]. Using vibration as an external stimulation is favorable particularly when controlling the motion of droplets since the acceleration is tunable depending upon droplet volumes, vibration amplitudes, and frequencies. An alternating current (AC) electrowetting-on-dielectric based platform was also used to manipulate a droplet on a ratchet surface [18]. In this platform, a droplet was manipulated since a voltage to an electrode converts the surface into a hydrophilic state, and attracts the droplet. The direction of the droplet motion on both Leidenfrost and vibration or AC-stimulated ratchet surfaces are dependent on the preset surface structure design and droplet size.

Over the above studies, more recent work presented a superhydrophobic black silicon ratchet surface on a magnetically actuated elastomer pillar array where the direction of the ratchet is dynamically controlled to manipulate droplets on it [19]. However, the uniformly actuated ratchet surface area was inherently limited since the magnetic force was induced because of the ferromagnetic iron particles embedded inside the elastomer pillars. Therefore, to form the ratchet, an external magnetic field must be applied in the direction perpendicular to the elastomer pillars such that each pillar experiences a different magnetic force.

**Figure 1:**
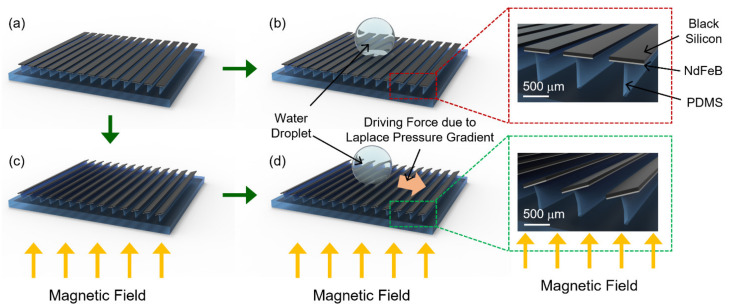
Illustrations of the magnetically actuated superhydrophobic black silicon strip array on an elastomer ridge array. (**a**,**b**) These illustrations show where no external magnetic field exists and thus no driving force is applied to a dispensed water droplet. (**c**,**d**) These illustrations show where an external magnetic field is applied, the strips are tilted, and a dispensed water droplet experiences a Laplace pressure gradient-induced driving force.

In this work, to resolve the inherent limit of the previously reported magnetically actuated ratchet surface, we employed a new fabrication strategy that creates a thin (20 µm thick) strip array with a black silicon on the front side and a neodymium-iron-boron (NdFeB) magnet on the back side, as depicted in Figure 1. Here, a NdFeB magnet is directly sputtered beneath the black silicon surface and magnetized in a direction parallel to the surface. Subsequently, the strip array is assembled on an elastomer ridge array via transfer printing to finally form the magnetically actuated ratchet surface in this work. Due to this fabrication strategy, an external magnetic field can be applied either upward or downward to create a more even magnetic torque in a large area, such that a spatially more uniform ratchet surface is formed. The detailed fabrication procedure, magnetomechanical model, and fluidic response of the reported superhydrophobic black silicon ratchet surface are discussed in the following sections.

## 2. Materials and Methods

### 2.1. Fabrication of an Elastomer Ridge Array

**Figure 2:**
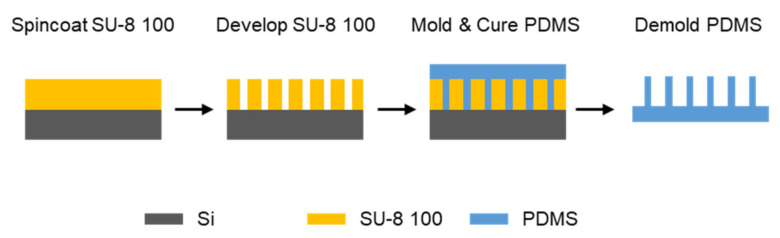
Fabrication of an SU-8 mold and an elastomer ridge array.

An array of polydimethylsiloxane (PDMS) elastomer ridges was fabricated by the following molding method. Each ridge is 50 µm wide, 200 µm high, and 10 mm long; the gap between ridges is 700 µm. First, as illustrated in Figure 2, an epoxy-based photoresist (SU-8 100, MicroChem Corp.) was disposed on a silicon wafer (UniversityWafer, Inc.). The resist was then spin-coated in two steps including spreading at 500 rpm for 5 s after 100 rpm/s acceleration, and then spinning at 3000 rpm for 20 s after 300 rpm/s acceleration. Next, the resist was soft-baked at 65 °C and 95 °C for 10 and 30 min, respectively, and cooled down to 40 °C to minimize any thermal stress. For the desired thickness, the above disposing, spin-coating, and soft-baking steps were repeated once more. The resist under a photomask was then exposed to ultraviolet light using a flood exposure tool (Model 60, ABM, Inc.) with an exposing dose of 600 mJ/cm^2^. The resist was post exposure baked at 65 °C and 95 °C for 1 and 20 min, respectively, and cooled down to 40 °C. The resist was developed in the developer (SU-8 developer, MicroChem Corp.) to fully remove the unexposed part. Once the resist was fully developed, the SU-8 mold was rinsed with isopropyl alcohol (IPA) and dried with gentle nitrogen flow.

To facilitate the releasing of PDMS from the SU-8 mold, trichloro(1H, 1H, 2H, 2H-perfluorooctyl)silane (Sigma-Aldrich) was deposited onto the SU-8 mold surface. The mold was first treated with oxygen plasma with RIE (Jupiter III, March instruments) for 1 min in a chamber of 50 mTorr with an oxygen flow rate of 20 sccm. Then, several trichlorosilane drops were placed in a vacuum jar with the mold, and the jar was pumped for 2 h. This process is hereafter called silane coating. After the silane coating, a PDMS precursor was mixed with 10:1 base to curing agent ratio, degassed, poured onto the SU-8 mold, and degassed again. Once degassing was complete, the PDMS precursor on the SU-8 mold was placed and cured in a 60 °C heated oven for 12 h. After fully curing, the PDMS was peeled out from the SU-8 mold to form an array of elastomer ridges, and the array was used later in the transfer printing step.

### 2.2. Fabrication of Nanostructured Black Silicon Strips

**Figure 3:**
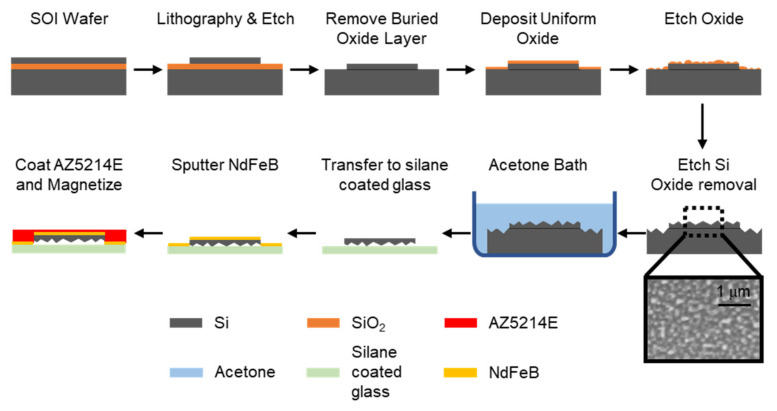
Fabrication of magnetized nanostructured black silicon strips.

As depicted in Figure 3, the 20 µm thick device layer of a silicon-on-insulator (SOI) wafer with a 1 µm thick buried oxide layer (Ultrasil LLC) was etched down to the buried oxide layer into an array of 500 µm wide, and 10 mm long strips with 250 µm gap in-between using an inductively coupled plasma deep reactive ion etcher (STS Pegasus) after a photolithography step. It is worth noting that these strips are tethered to a single square frame so that all strips are transferred simultaneously, which is explained in the next section. The procedure for creating a nanostructured silicon surface, called black silicon, follows [20]. First, the remaining resist was removed, and the wafer was placed in a hydrofluoric acid (HF, 48% concentration) bath for 17 h to clear off any native oxide on the silicon surface and the buried oxide layer. Then, a uniform layer of oxide was deposited using oxygen plasma (50 mTorr, O_2_ 10 sccm, RF1 120 W, RF2 200 W, 5 min, Plasmatherm ICP RIE). Next, the deposited oxide layer was etched to form randomly scattered oxide islands (50 mTorr, CHF_3_ 12 sccm, RF 350 W, 2 min). Lastly, the silicon with a scattered oxide hard mask was then etched with chlorine plasma, yielding a forest of nano conical structures (90 mTorr, Cl_2_ 40 sccm, Ar 4 sccm, RF1 300 W, RF2 500 W, 10 min). The remaining oxide was removed by placing the wafer into an HF bath. The resulting SEM image of black silicon surface can be found in Figure 3.

### 2.3. Transferring Black Silicon Strips and Sputtering a Magnetic Material

Continuing from the previous steps in Figure 3; once the nano conical structures were formed, the wafer was placed in an acetone bath to remove the array of black silicon strips from the handle layer of the wafer. Using a tweezer, the array was picked up and placed on a silane-coated glass slide with the nanostructures facing down. Next, the black silicon strips were inserted in a sputter machine (Kurt J. Lesker Company), and NdFeB was deposited for 40 min with RF power of 120 W. To carry it more stably, a photoresist (AZ5214E, MicroChemicals) was spin-coated on top of it. Finally, the NdFeB was magnetized in a 1.9 T field (Magnetic Instrumentation).

### 2.4. Transfer Printing of Black Silicon Magnetic Strips on Elastomer Ridges

**Figure 4:**
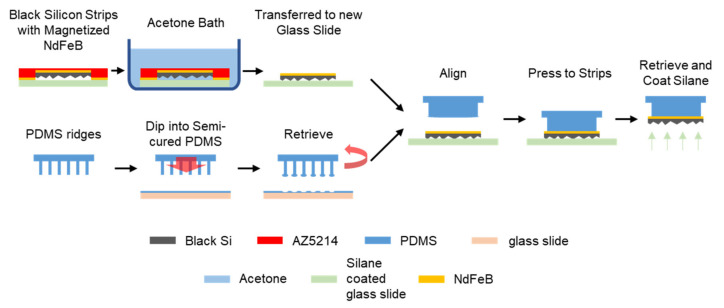
Transfer printing of the black silicon strips to the array of elastomer ridges.

As shown in Figure 4, the array of NdFeB deposited black silicon strips was transfer-printed to the array of elastomer ridges. As a first step of the transfer printing, the strips were immersed under an acetone bath to remove the photoresist. After 3 min, the strips were carefully transferred to a new silane-coated glass slide with nanostructures facing down, using tweezers. In the meanwhile, a 10:1 base to curing agent mixture of PDMS precursor was poured onto a clean glass slide, spin-coated at 5000 rpm for 30 s, and semi-cured for 15 min at 65 °C. Next, the elastomer ridges were dipped into the semi-cured PDMS, which formed a microbead on each end of the ridges. Then, the elastomer ridges were manually aligned with the strips using transitional and rotational stages (Karl Suss MJB3 mask aligner). Once the alignment was complete, the elastomer ridges were pressed to the strips and retrieved from the glass slide quickly to maintain the adhesion between the semi-cured PDMS and the strips. After the retrieval, the strips were released from the square frame by breaking the tethers of the strips. Finally, to make the nanostructured black silicon strips superhydrophobic, the silane coating procedure was performed on the surface.

### 2.5. High-Speed Camera Capturing

**Figure 5:**
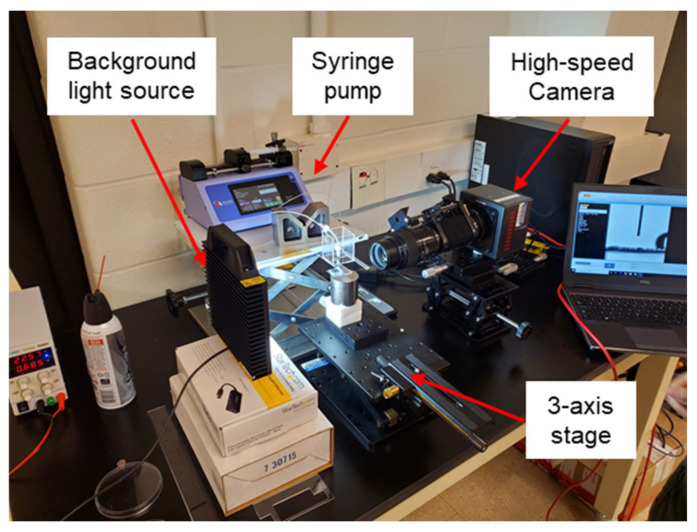
The test setup for capturing high-speed camera images.

The setup for capturing the motion of the water droplet and its contact angle is shown in Figure 5. The frame rates of the high-speed camera (FASTCAM Mini AX200, Photron) are 2000 fps for impact tests and 50–200 fps for spreading or contact angle measurements. The experiments were conducted at 1 atm and 20 °C. A background LED light source was provided for easier identification of the droplets, and the running time was less than 2 min for every experimental cycle, which was not expected to cause any temperature change. A syringe pump (11 Pico Plus Elite, Harvard Apparatus) and a blunt stainless steel needle were used for uniform dispensing of a water droplet. A testing ratchet surface, a magnet, and a camera were installed on different XYZ linear stages.

## 3. Results and Discussion

### 3.1. Characterization of Magnetomechanical Properties

**Figure 6:**
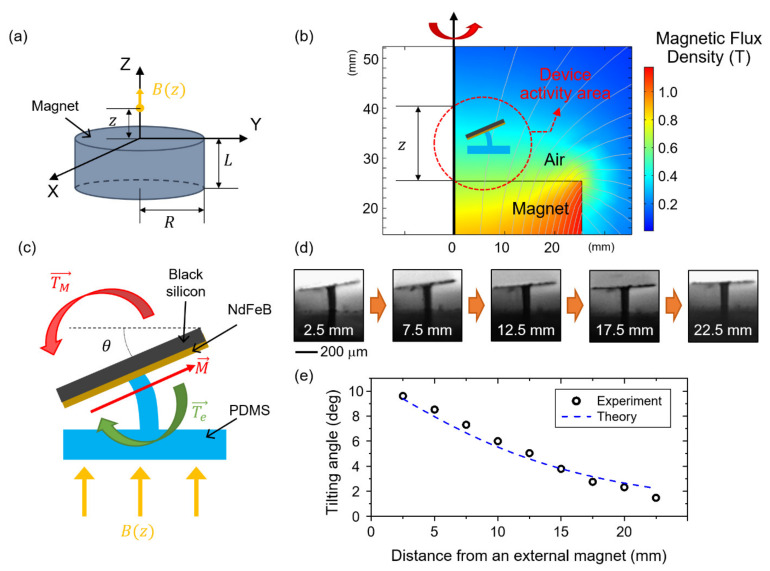
(**a**) The dimension of an external magnet where its z-axis magnetic flux density is indicated. (**b**) A fringe plot of a magnetic flux density from the external magnet. (**c**) Schematic illustration of two competing torques applied to a strip combined with an elastomer ridge. (**d**) Experimentally captured sequential photos of the tilted strip at different distances from the external magnet. (**e**) A graph of theoretically calculated and experimentally measured tilting angles as a function of the distance from the external magnet.

As illustrated in Figure 1, the magnetically actuated superhydrophobic black silicon strips are flat when an external magnetic field does not exist. Therefore, when a droplet is dispensed, it does not move since there is no driving force. Once a magnetic field is applied to the strips, a magnetic torque is generated, and the strips are tilted with a tilting angle of θ as represented in Figure 6c. The non-zero tilting angle causes the asymmetric shape of a dispensed droplet and, resultantly, a Laplace pressure gradient inside, which will be explained later.

In order to theoretically calculate the tilting angle, the magnitude of the magnetic flux density is calculated. The z-direction magnetic flux density $B\left(z\right)$ along the center axis of the external cylindrical magnet is defined as

(1) $$B\left(z\right)=\frac{B_{r}}{2}\left(\frac{z+L}{\sqrt{R^{2}+{\left(z+L\right)}^{2}}}-\frac{z}{\sqrt{R^{2}+z^{2}}}\right)$$

where $B_{r}$ is the remanent flux density of the external magnet, z is the distance from the magnet surface, and L and R are thickness and radius of the external magnet, respectively, as illustrated in Figure 6a [21]. In addition, the numerically analyzed magnetic flux density is plotted in Figure 6b (COMSOL Multiphysics). Here we chose $B\left(z\right)$ along the center axis of the external cylindrical magnet since the magnetic flux density gradient in the z-direction varied at most 7%, regarding the radial location within the device activity area. Thus, the tilting angle is investigated as a function of the vertical distance between the external magnet and the strip in this work.

Assuming that the strips are in equilibrium with θ, there exists two competing torques that negate each other [19]. One is the magnetic torque *T_m_* which can be expressed as $T_{m}=\mu_{0}\vec{m}\times \vec{H}$. $\mu_{0}$ is a vacuum permeability and m is a magnetic dipole moment for the sputtered NdFeB layer, which can be obtained from $\vec{m}=\vec{M}V_{m}$ where *M* is a magnetization and *V_m_* is the volume of the NdFeB layer. The magnetic field $\vec{H}$ is converted to $\vec{B\left(z\right)}/\mu_{0}$ for Equation (1). The other one is an elastic restoring torque $T_{e}=K_{eq}\theta$. $K_{eq}$ is the equivalent torsion spring constant, which can be approximated as $K_{eq}=cEI/L$, where c is a correction coefficient; *E*, *I*, and *L* are the elastic modulus, second moment of inertia, and height of the PDMS ridge, respectively. In equilibrium, the two torques balance each other with *T_m_* = *T_e_* and therefore the tilting angle θ can be expressed as

(2) $$\theta =\frac{MV_{m}B\left(z\right)}{K_{eq}}\cos\theta$$

Here, the *cos θ* came from the cross product of *M* and *B(z)*. Equation (2) is numerically solved using MATLAB, and the calculated tilting angle (*θ*) is plotted in Figure 6e.

To confirm the numerical results, the tilting angle (*θ*) is also experimentally measured. Figure 6d depicts each step of angle change of the same strip as a function of the distance from the external magnet. The tilting angle at *z* = 2.5 mm is 9.605°, and the tilting angle steadily decreases as the strip moves away from the external magnet and reaches 1.474° at *z* = 22.5 mm Here, the angles are measured using ImageJ software five times each, and averaged. These discrete image results are also summarized in Figure 6e.

### 3.2. Droplet Spreading Behavior

**Figure 7:**
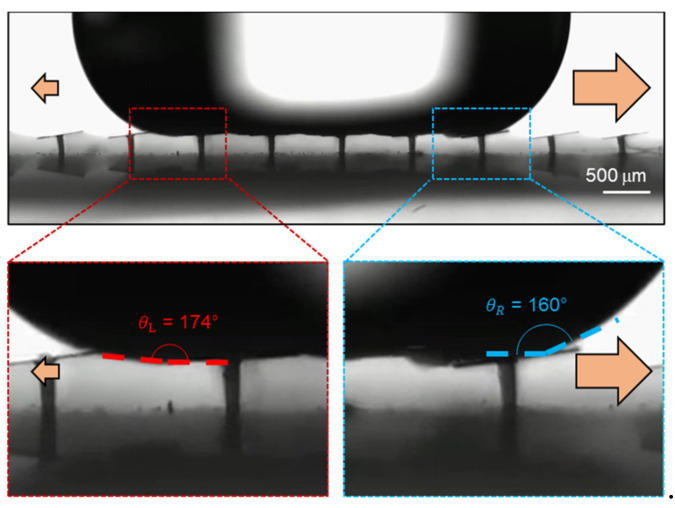
The two different contact angles of a water droplet with a volume of 17.4 μL on a black silicon ratchet surface.

If there is no other external force, such as gravity, the contact angle of an asymmetric droplet controls the net driving force when a droplet is placed on a surface [22,23]. The relationship below expresses the force that drives a droplet from left to the right, which involves the Laplace pressure gradient caused by the asymmetric droplet shape.

(3) $$F_{L}\sim R_{0}\gamma \left(\cos\theta_{R}-\cos\theta_{L}\right)$$

where *R*_0_, *γ*, *θ_R_*, and *θ_L_* are the radius, the surface tension, and right and left side contact angle of a droplet, respectively. In the situation where there is no magnetic field, as shown in Figure 1b, the surface is flat and thus the droplet is symmetric (*θ_R_* = *θ_L_*). In this case no droplet motion is observed since the driving force is zero. When an external magnetic field is applied, the strip surface forms a ratchet shape resulting in an asymmetric droplet shape (*θ_R_* < *θ_L_*). The actual left and right contact angles of a deionized (DI) water droplet, on the superhydrophobic black silicon ratchet surface when spreading, were measured in Figure 7 where *θ_L_* and *θ_R_* are 174° and 160°, respectively. Since (*cos θ_R_* − *cos θ_L_*) > 0 in Equation (3), the Laplace pressure gradient-induced driving force is applied towards right as indicated in Figure 1d. Here, it is worthwhile to note that *θ_L_* = θ* − Δ*θ* = 167° − 7°and *θ_R_* = θ* + Δ*θ* = 167° + 7° where θ* is the advancing contact angle of a DI water droplet on the flat strip surface with no external magnetic field, and Δ*θ* is the tilting angle of the black silicon strips.

**Figure 8:**
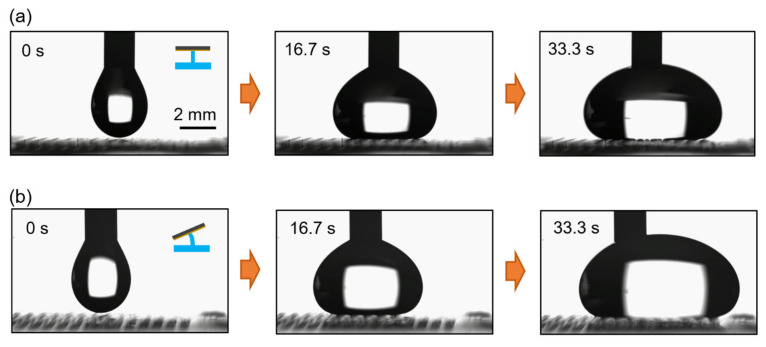
(**a**) Symmetric spreading behavior of a water droplet when no magnetic field is applied and black silicon strips are flat. (**b**) Asymmetric spreading behavior of a water droplet when a magnetic field is applied and black silicon strips form a ratchet surface.

When dispensed on a superhydrophobic flat or ratchet surface, a water droplet is in a symmetric or asymmetric shape, respectively (Figure 1b,d). The related droplet spreading characteristics were investigated and summarized in Figure 8. From a needle, a water droplet is dispensed at the flow rate of 50 µL/min. An external magnetic field is not applied, and thus the black silicon strips are flat in Figure 8a. Since the water droplet shape is symmetric, and any related forces balance out, the water droplet spreads symmetrically. On the other hand, once an external magnetic field is applied, the strips are tilted to form the black silicon ratchet surface, and the water droplet shape is asymmetric. Consequently, the force by the Laplace pressure gradient induces an uneven spreading of the water droplet as depicted in Figure 8b.

### 3.3. Droplet Impact Behavior

**Figure 9:**
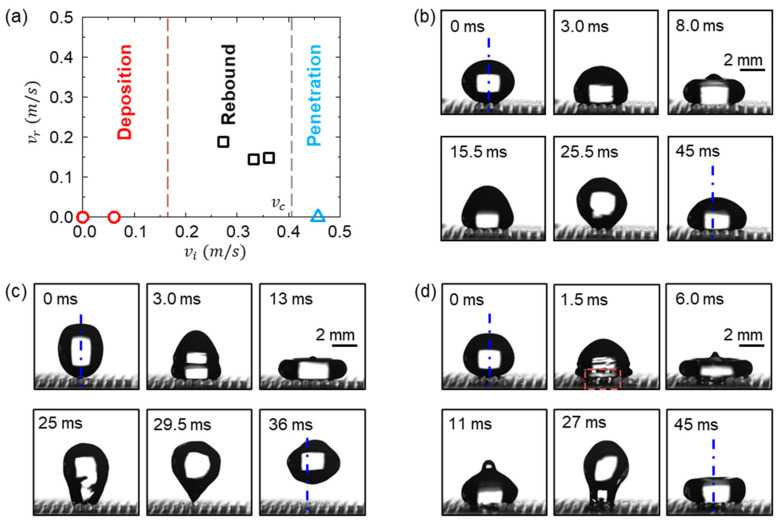
(**a**) Rebound velocities (*v_r_*) of water droplets with a diameter of *D* = 3.95 mm impacting a magnetically actuated superhydrophobic ratchet surface with different impact velocities (*v_i_*). Sequential images of the droplet impact processes showing different behaviors of (**b**) deposition with *v_i_* = 0.06 m/s and *We* = 0.2, (**c**) rebound with *v_i_* = 0.27 m/s and *We* = 4.0, and (**d**) penetration with *v_i_* = 0.46 m/s and *We* = 11.3. The measurement error for the velocity is ≤ 0.013 m/s and the relative repeating error is ≤10.5%. The tilting angle of the strips is about 8°. The blue dash-dotted line represents the axis of the droplet before impact. The red highlighted zone shows the penetration of the impacting droplet into the grooves between the strips.

Figure 9 shows the impact behaviors of water droplets with diameters of *D* = 3.95 mm onto the magnetically actuated black silicon ratchet surface, where the tilting angle of the strips is about 8° under an external magnetic field. When the impact velocity *v_i_* increases from 0 to 0.46 m/s, three impact behaviors of water droplets were observed, i.e., deposition, rebound, and penetration. Figure 9a relates three impact behaviors with the impact velocity (*v_i_*), and Figure 9b–d shows their typical dynamic features.

After impacting the ratchet surface, the droplet first undergoes the spreading stage (the first row in Figure 9b–d) where the contact line advances continuously with a contact angle larger than 160°, which is similar to those of the impact droplets on continuous flat superhydrophobic surfaces [24,25,26,27]. The droplet deforms into a pancake-like shape when the contact line velocity decreases to zero, with its maximum spread radius increased with *v_i_*. Then, the retraction stage occurs where the contact line recedes, and the droplet tends to recover its original shape (the second row in Figure 9b–d). In contrast to the impact on a continuous flat surface, a “stick-jumping” phenomenon is observed during the retraction stage, which is caused by the pinning between the droplet and the side edge of the strip. This phenomenon is also observed in the previous studies of a droplet impacting on the grooved surfaces [28,29]. The receding contact angle during the retraction stage is much smaller than the advancing contact angle during the spreading stage. The droplet cannot bounce off the surface when *v_i_* is small because the kinetic energy is less than the surface energy dissipated during the retraction stage, showing the deposition behavior. Figure 9b shows this deposition behavior when *v_i_* = 0.06 m/s and *We* = 0.2, where *We = ρv_i_^2^D/γ* with *ρ* as the water density and *γ* as the water surface tension, which compares the inertia with the surface tension forces. Increasing *v_i_* enlarges the kinetic energy of the droplet, which finally overcomes the surface energy dissipated during impact and makes the droplet bounce off, exhibiting the rebound behavior. This rebound behavior of the droplet with *v_i_* = 0.27 m/s and *We* = 4.0 is demonstrated in Figure 9c. The criterion between the deposition and rebound can be obtained by balancing the initial kinetic energy of the droplet and the dissipated surface energy during the retraction stage, yielding a critical Weber number of *We_cr_* ~ *O*(1) [30] and a critical impact velocity of *v_cr_ ≈* 0.14 m/s, which is consistent as the experimentally determined boundary between the deposition and rebound behaviors in Figure 9a. Remarkably, at the early spreading stage (1.5 ms in Figure 9d), the penetration of the droplet was observed through the grooves between the strips when *v_i_* = 0.46 m/s with *We* = 11.3. The adhesion of the droplet in the grooves increases the surface energy change, and thus suppresses the rebound of the droplet during the retraction stage, which is denoted as the penetration behavior.

Unlike the impact on a flat strip surface, the droplet exhibits a directional lateral motion when impacting on the ratchet surface. As shown in Figure 9c, due to the Laplace pressure gradient (Equation (3)), the droplet shifts its position towards the right, compared to its original position when bouncing off the surface. This indicates that the surface exhibits directional wettability [29,31] when the strips are tilted. The directional droplet bouncing demonstrates the potential of the reported ratchet surface in the manipulation of the droplet impact dynamics, using a controllable magnetic field for practical situations. 

However, it should be noted that the penetration of the droplet at a higher *v_i_* largely suppresses the directional motion of the impacting droplets, as shown in Figure 9d, where the droplet essentially maintains its original position during the impact. The occurrence of droplet penetration indicates the change of the droplet wetting state from the Cassie state towards the Wenzel state, with the decrease in the surface hydrophobicity. The transition of the wetting stage occurs when the wetting pressure of the droplet *P_D_* overcomes the anti-wetting pressure (capillary pressure) of the grooves *P_C_*, where *P_D_ ~ ρv_i_*^2^ and *P_C_ ~* *γcos*θ*/*w_g_* with *w_g_* as the groove width between the strips [30,32]. Then, when *P_D_* ≈ *P_C_*, we obtain a critical impact velocity of *v_cp_* ≈ 0.53 m/s. The predicted velocity is also close to the experimentally determined boundary between the rebound and penetration behaviors in Figure 9a. In addition, it is noted that decreasing *w_g_* can increase the critical impact velocity to avoid droplet penetration. Therefore, further work will be dedicated to shortening the distance between strips to maintain the robust functionality of the magnetically actuated black silicon ratchet surface.

## 4. Conclusions

In conclusion, this work presents a magnetically actuated superhydrophobic ratchet surface composed of nanostructured black silicon strips and investigates their magnetomechanical characteristics and fluidic responses. The tilting angle of the strips calculated with the theoretical model matched the experimentally measured one ranging from 9.605° to 1.474°, depending on the distance between the strips and an external magnet. The tilted strips formed a superhydrophobic ratchet shape surface over which a droplet showed asymmetric spreading. Furthermore, the droplet impact test was performed, and three different responses were observed. A critical impact velocity of droplet penetrating into the gap between the strips was calculated, which can be increased by decreasing the gap between the strips to improve their robustness for droplet manipulation. The surface quality of the device did not degrade after repeated tests; however, quantitative research on durability will be conducted for future work.
